# Research progress and advances in endoplasmic reticulum stress regulation of acute kidney injury

**DOI:** 10.1080/0886022X.2024.2433160

**Published:** 2024-11-25

**Authors:** Li-Ran Zhu, Wei Cui, Hai-Peng Liu

**Affiliations:** a Anhui Institute of Pediatric Research, Anhui Provincial Children’s Hospital (Children’s Hospital of Fudan University Anhui Hospital; Children’s Medical Center of Anhui Medical University), Hefei, Anhui, China; b Department of Scientific Research and Education, Anhui Provincial Children’s Hospital (Children’s Hospital of Fudan University Anhui Hospital; Children’s Medical Center of Anhui Medical University), Hefei, Anhui, China

**Keywords:** Acute kidney injury, endoplasmic reticulum, signaling pathway, therapeutic approach

## Abstract

Acute kidney injury (AKI) is a common and severe clinical disorder in which endoplasmic reticulum (ER) stress plays an important regulatory role. In this review, we summarize the research progress on the relationship between ER stress and AKI. It emphasizes the importance of maintaining a balance between promoting and protecting ER stress during AKI and highlights the potential of ER stress-targeted drugs as a new therapeutic approach for AKI. The article also discusses the need for developing drugs that target ER stress effectively while avoiding adverse effects on normal cells and tissues. The review concludes that with a more comprehensive understanding of ER stress mechanisms and advancements in research techniques, more effective treatment options for AKI can be developed in the future.

## 1. Introduction

AKI is a prevalent clinical condition characterized by a rapid decline in renal function over a short period of time, resulting in metabolic disturbances such as reduced urine output, azotemia, uremia, and metabolic acidosis [1]. Severe cases of AKI may require renal replacement therapy, such as dialysis, and can even lead to patient death [2,3]. Endoplasmic reticulum stress (ER stress) has been found to be closely associated with AKI [4]. The ER is a vital structural system of membranes within cells that is responsible for protein folding, modification, and transport. When the ER function is impaired, it results in ER stress [5].

AKI is a pathological state in which renal function decreases dramatically within a short period of time and may be caused by various factors, including ischemia, toxicity, and infection. ER stress plays a significant role in the occurrence and development of AKI [6]. Renal cells are subjected to stimuli such as ischemia, hypoxia, and inflammation when AKI occurs, leading to increased ER stress. ER stress activates a series of cellular stress responses, such as the unfolded protein response (UPR), to preserve ER function and restore cellular homeostasis [7]. However, prolonged ER stress can impair cellular function, contributing to apoptosis and renal function deterioration. Thus, the relationship between ER stress and AKI is bidirectional.

This review article sheds light on the crucial relationship between ER stress and AKI. It emphasizes the importance of maintaining balance between promoting and protecting ER stress during AKI, and it outlines the potential of ER stress-targeted drugs as a new therapeutic approach for AKI. Overall, this review provides a comprehensive understanding of the intricate relationship between AKI and ER stress and offers a foundation for future research in this area.

## 2. Classification, characteristics, and treatment strategies of AKI

### 2.1. Etiology and classification of AKI

#### 2.1.1. Etiology

AKI is a common complication among hospitalized patients globally, with an incidence of approximately 1–2%, which can rise to as high as 50% in critically ill patients, particularly those in intensive care units (ICUs)[8]. The incidence may be higher in developing countries with limited healthcare resources. AKI is triggered by various factors, including ischemic, nephrotoxic, infectious, inflammatory, and obstructive causes [9]. Common risk factors include hypertension, diabetes, cardiovascular disease, advanced age, specific medications (e.g., NSAIDs, antibiotics, and contrast agents), and surgical interventions. Critically ill and ICU patients are especially susceptible to AKI. Additionally, genetic factors, ethnic differences, and socioeconomic factors may also impact AKI incidence.

#### 2.1.2. Classification

AKI is generally classified into three main types: prerenal, intrinsic, and postrenal. Prerenal AKI results from inadequate blood flow to the kidneys, intrinsic AKI is caused by direct damage to renal tissues, and postrenal AKI is due to urinary obstruction or reflux. While this classification helps in understanding the etiology of AKI, the primary focus of this review is on the molecular mechanisms, particularly the role of ER stress. ER stress plays a critical role in the pathogenesis of AKI, affecting cellular responses in all three types. In postrenal AKI, ER stress is particularly involved in modulating repair mechanisms and influencing cellular survival [10,11]. However, excessive ER stress can lead to apoptosis, worsening renal injury. This underscores the dual role of ER stress in both the progression of AKI and its potential as a therapeutic target [12].

### 2.2. Diagnosis and treatment strategies for AKI

In recent years, diagnostic criteria for AKI have been progressively refined. Biomarkers such as creatinine and urine output help assess the severity of AKI. The KDIGO (Kidney Disease: Improving Global Outcomes) clinical practice guidelines are now widely recognized standards for AKI diagnosis and staging, enhancing understanding and early intervention capabilities for AKI [13]. In addition to creatinine and urine output, several novel biomarkers, including kidney injury molecule-1 (KIM-1) and neutrophil gelatinase-associated lipocalin (NGAL), have been developed to aid in early detection [14–16].

**Nephrotoxic drug-induced AKI** is common in clinical settings and involves a range of drugs, such as certain antibiotics, NSAIDs, chemotherapeutic agents, and contrast agents. These drugs can directly damage renal cells, resulting in declining kidney function. *In vitro* studies reveal mechanisms through which these drugs induce renal injury and suggest potential protective strategies. For example, nephrotoxic drugs have been shown to increase intracellular oxidative stress and activate ER stress pathways, ultimately leading to programmed cell death [17,18]. Understanding these mechanisms helps elucidate the molecular basis of drug-induced renal damage, providing insights for developing preventive and therapeutic strategies. Additionally, some *in vitro* studies have evaluated the potential protective effects of specific compounds or drugs against nephrotoxic drug-induced AKI, finding that certain antioxidants, molecular chaperone enhancers, or specific signaling pathway inhibitors may alleviate drug-induced cellular damage [19,20].

Currently, AKI treatment focuses on addressing the etiology and pathophysiological processes involved. Common therapeutic measures include: (1) **Treating the cause**: For instance, ischemic AKI requires restoration of blood flow, nephrotoxic AKI requires discontinuation of nephrotoxic drugs, and infectious AKI requires antibiotic treatment. (2) **Maintaining water and electrolyte balance**: Adjusting infusion types and amounts based on the patient’s urine output, cardiac, and renal function; monitoring blood volume, sodium, potassium, and acid-base balance to correct imbalances promptly. (3) **Dialysis and renal replacement therapy**: Patients with uremia, severe edema, critical electrolyte, and acid-base imbalances, or toxicity uncorrectable by conservative therapy require dialysis or other renal replacement therapies. (4) **Renal protection strategies**: Avoid nephrotoxic drugs, adjust drug doses to prevent accumulation, limit contrast agent use, and maintain proper blood pressure and cardiac output. (5) **Nutritional support**: Provide appropriate calories, proteins, vitamins, and micronutrients according to the patient’s nutritional status and metabolic needs. (6) **Prevention of complications**: Prevent infections, ulcers, and pulmonary complications [2,21].

## 3. ER stress process and corresponding pathways

### 3.1. Key stages of ER stress

The ER is a crucial organelle in cells, responsible for protein synthesis, folding, modification, and calcium storage [22]. ER stress arises when these functions are disrupted, causing an accumulation of misfolded or unfolded proteins and triggering a cellular stress response known as the UPR [23,24]. Triggers of ER stress include gene mutations, oxidative stress, viral infections, diabetes, malnutrition, and drug toxicity.In response to these stressors, ER sensors—PERK(Protein Kinase RNA-like Endoplasmic Reticulum Kinase), IRE1(Inositol-Requiring Enzyme 1), and ATF6 (Activating Transcription Factor 6)—detect the build-up of unfolded proteins, activating the UPR to reduce ER burden through mechanisms like reduced protein synthesis, enhanced protein folding, and increased degradation of misfolded proteins [25–28]. In response to these stressors, ER sensors—PERK, IRE1, and ATF6—detect the build-up of unfolded proteins, activating the UPR to reduce ER burden through mechanisms like reduced protein synthesis, enhanced protein folding, and increased degradation of misfolded proteins [29].

### 3.2. Cellular responses to ER stress: repair vs. apoptosis

ER stress plays dual roles in cell fate, exerting both protective and harmful effects depending on stress levels. Under moderate stress, ER stress promotes autophagy, a mechanism to degrade abnormal proteins, maintain homeostasis, and enhance cell survival [30–32]. However, prolonged ER stress results in elevated intracellular calcium and sustained UPR activation, leading to mitochondrial dysfunction and apoptosis as a protective measure to eliminate severely damaged cells [33]. Thus, the UPR initially promotes cellular adaptation but shifts to apoptosis if stress persists [34–37].

### 3.3. ER stress pathways in AKI pathogenesis and experimental models

The three major UPR pathways—IRE1, PERK, and ATF6—play critical roles in regulating cellular responses during ER stress and influence AKI pathogenesis. Experimental models of AKI, particularly nephrotoxic and ischemia-reperfusion injury (IRI) models, have shown how these pathways contribute to disease progression and highlight their potential as therapeutic targets.

IRE1 Pathway: IRE1 regulates protein folding and degradation by activating XBP1, a transcription factor that modulates adaptive responses to ER stress [38]. In nephrotoxic AKI models, IRE1 has shown to mitigate oxidative stress and inflammation by reducing unfolded protein accumulation and moderating JNK activation, a key driver of apoptosis in kidney cells. However, prolonged activation of IRE1 can promote pro-inflammatory responses, potentially exacerbating kidney injury. Studies suggest that targeting IRE1 may reduce oxidative damage and inflammation, offering protection against AKI in models of drug-induced nephrotoxicity [39].

PERK Pathway: PERK reduces protein synthesis by phosphorylating eIF2α, decreasing ER load. In AKI experimental models, PERK has demonstrated protective roles by activating antioxidant pathways, particularly the nuclear factor erythroid 2-related factor 2 (Nrf2), which defends against oxidative stress, a key factor in AKI pathogenesis [40]. However, prolonged PERK activation upregulates CHOP, a pro-apoptotic protein that triggers cell death, contributing to renal injury under chronic stress. Modulating PERK activity, especially in ischemic AKI models, has shown potential in reducing renal injury and preventing apoptosis, highlighting the pathway’s therapeutic promise [41].

ATF6 Pathway: ATF6 supports cellular adaptation by enhancing the ER’s protein-folding capacity and upregulating stress-related genes [42]. AKI models have demonstrated that ATF6 activation aids in cellular repair and recovery during early injury phases, promoting cell survival. However, chronic ATF6 activation can intensify inflammatory responses, worsening renal damage under prolonged stress conditions. Experimental studies exploring ATF6 modulation suggest its potential to improve cellular resilience in AKI by reducing protein misfolding and boosting cellular repair mechanisms [43].

**Figure 1.**
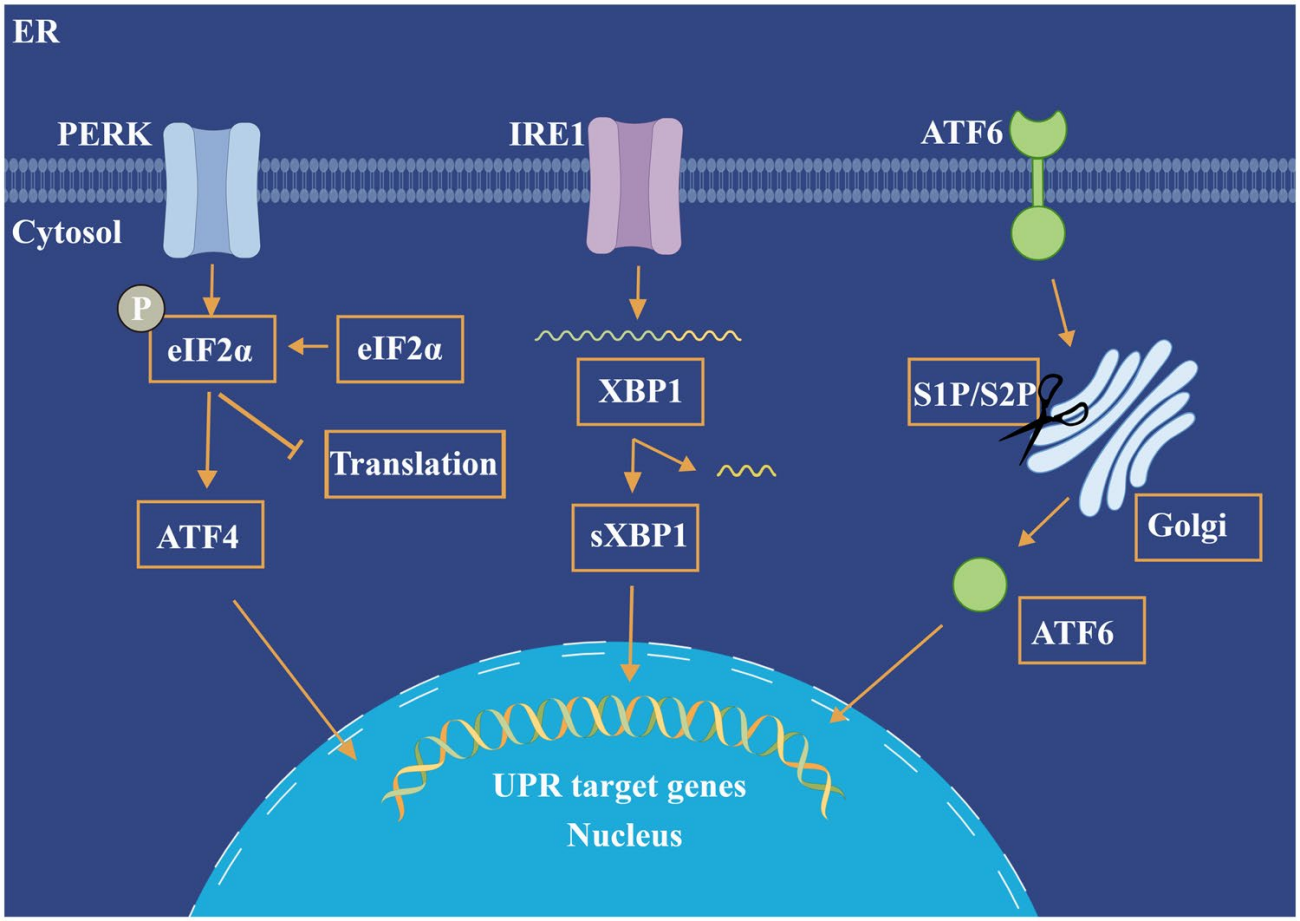
ER stress pathways in the unfolded protein response (UPR). This figure illustrates the three main ER stress response pathways: **PERK** (Protein Kinase RNA-like Endoplasmic Reticulum Kinase), **IRE1** (Inositol-Requiring Enzyme 1), and **ATF6** (Activating Transcription Factor 6). PERK phosphorylates **eIF2α** (Eukaryotic Initiation Factor 2α) to reduce protein synthesis and activates ATF4 to promote stress adaptation. IRE1 splices **XBP1** mRNA, producing sXBP1, which enhances UPR target genes. ATF6 is cleaved in the Golgi and moves to the nucleus to boost protein-folding capacity. These pathways collectively alleviate ER stress by balancing protein synthesis, folding, and degradation

By using these experimental AKI models, researchers have highlighted the dual potential of UPR pathways as both adaptive and pathological factors in AKI. This suggests that selectively modulating IRE1, PERK, or ATF6 may allow for a balanced approach, enhancing protective responses while inhibiting harmful ones. These findings underscore the clinical potential of targeting specific UPR pathways to mitigate AKI progression and prevent cell death [44,45] (Figure 1).

### 3.4. Therapeutic approaches targeting ER stress in AKI: clinical translation potential

Therapies targeting ER stress pathways offer promising new approaches for AKI management [46–48]. Chemical chaperones like tauroursodeoxycholic acid (TUDCA) and 4-phenylbutyric acid (4-PBA) reduce ER stress by promoting protein folding and aiding in the clearance of misfolded proteins, helping to alleviate cellular stress [49]. Proteasome inhibitors (e.g., Bortezomib) and autophagy inducers (e.g., Rapamycin) also reduce cellular stress load by removing protein aggregates and promoting cellular recovery [50,51]. However, stress-inducing agents like Thapsigargin and Tunicamycin, which increase ER stress, highlight the need for precise modulation to avoid excessive stress responses that could exacerbate kidney injur [52,53]. Careful selection and application of ER stress-targeting therapies are crucial, as improper use may intensify renal damage rather than alleviate it. These preclinical models and experimental therapies indicate that UPR modulation holds significant translational potential in AKI treatment, though patient-specific adjustments are essential to avoid adverse outcomes.

## 4. The relationship between ER stress and AKI

### 4.1. ER stress in ischemia-reperfusion injury (IRI)

IRI is a common cause of AKI in clinical contexts such as renal transplantation, shock, and cardiovascular surgery. The injury process involves complex molecular and cellular mechanisms during hypoxia and subsequent reperfusion. ER stress plays both protective and detrimental roles in IRI, regulating a dynamic balance that significantly impacts the outcome of the injury [5].

#### 4.1.1. Protective effects

In IRI, ER stress exerts protective effects through multiple pathways that help reduce cell damage associated with ischemia and reperfusion. First, ER stress enhances protein folding capacity by upregulating molecular chaperones and folding enzymes *via* the PERK and ATF6 pathways, facilitating the proper folding of accumulated unfolded proteins during ischemia [25,26]. The PERK pathway also reduces the ER burden by phosphorylating eIF2α, which decreases overall protein synthesis [27]. Additionally, the ATF6 pathway modulates inflammation by inhibiting the NF-κB (Nuclear Factor Kappa B) signaling pathway, thereby reducing the production of inflammatory cytokines [28,54]. Concurrently, the PERK pathway induces Nrf2 (Nuclear Factor Erythroid 2–Related Factor 2) expression, promoting the production of antioxidant enzymes to protect cells from oxidative damage [29,32]. ER stress further promotes cell survival by enhancing cell cycle protein and growth factor expression, supporting cell proliferation and tissue regeneration [31]. Through the induction of autophagy-related genes, ER stress also aids in the clearance of intracellular waste, maintaining cellular homeostasis [33–37].

#### 4.1.2. Detrimental effects

While ER stress provides some protection, excessive ER stress may lead to cell apoptosis and other harmful effects. Overactivation of the PERK pathway significantly upregulates CHOP (C/EBP Homologous Protein), which promotes apoptosis by inhibiting Bcl-2 proteins and activating the Bax/Bak pathway, thereby exacerbating mitochondrial dysfunction and causing cell death [55]. In cases of severe ER stress, NF-κB activation increases the production of inflammatory cytokines like TNF-α, IL-1β, and IL-6, inducing apoptosis through the JNK and p38 MAPK pathways [38,39,56]. ER stress also disrupts calcium homeostasis, leading to mitochondrial dysfunction and excessive reactive oxygen species (ROS) production, which further drives oxidative stress [37]. The IRE1α pathway activates JNK signaling and caspase-12, which subsequently induces apoptosis [56]. Furthermore, excessive ER stress can lead to impaired autophagic flux, resulting in inefficient degradation of autophagosomes and promoting apoptosis [38]. Disruption of ER-mitochondrial contact sites affects calcium signaling and mitochondrial function, contributing to increased cell death [39]. This imbalance also disrupts energy metabolism, reducing ATP production and glycolysis, leading to energy deficits and cell death [40].

**Figure 2.**
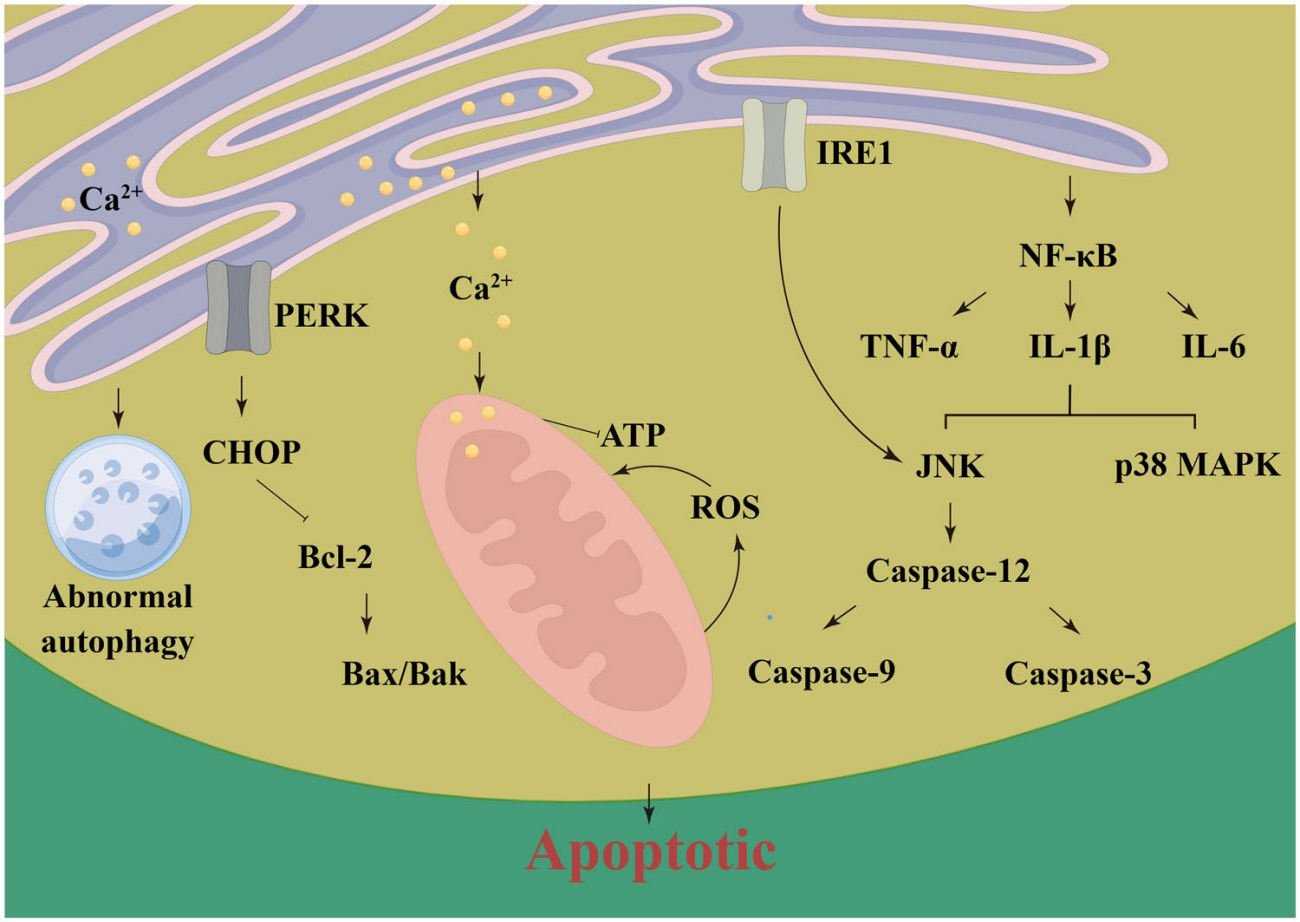
Mechanisms of ER stress-induced apoptosis in acute kidney injury (AKI). This figure shows how ER stress can lead to apoptosis via **PERK** and **IRE1** pathways. PERK activates **CHOP** (C/EBP Homologous Protein), which promotes apoptosis by regulating **Bcl-2** and **Bax/Bak**. IRE1 activates **NF-κB** (Nuclear Factor Kappa B), increasing inflammatory cytokines (**TNF-α**, **IL-1β**, **IL-6**) and triggering **JNK** and **p38 MAPK** pathways, which lead to apoptosis through caspase activation. ER stress-induced calcium release also causes mitochondrial dysfunction and increased **ROS** production, further driving apoptosis.

Understanding the balance between the protective and detrimental effects of ER stress in IRI is essential for developing effective therapeutic strategies. Future treatments could focus on modulating ER stress to maintain an optimal stress level that maximizes protective effects while minimizing harmful outcomes (Figure 2).

### 4.2. Regulation of the dynamic balance with nephrotoxic AKI

Nephrotoxic AKI is primarily caused by drugs, toxins, or other exogenous factors. ER stress significantly influences cellular responses to nephrotoxins, playing both protective and damaging roles.

#### 4.2.1. Protective effects

In nephrotoxic AKI, ER stress mitigates cellular damage by enhancing protein folding through upregulation of molecular chaperones like protein disulfide isomerase (PDI) and heat shock proteins (HSPs)[27]. The activation of the ER-associated degradation (ERAD) pathway aids in degrading misfolded proteins, alleviating ER burden, and improving cell survival [46]. The PERK pathway further suppresses NF-κB signaling, reducing inflammation in response to nephrotoxins [48]. Additionally, ER stress activates the Nrf2/ARE pathway, which induces antioxidant enzymes to counter oxidative injury [39]. ER stress also supports repair and regeneration of damaged renal cells by upregulating growth factors and cell cycle proteins [46]. ER stress can regulate extracellular matrix synthesis and secretion, facilitating renal tubular remodeling by modulating matrix metalloproteinases (MMPs) and tissue inhibitors of metalloproteinases (TIMPs)[47]. Through autophagy and mitochondrial quality control, ER stress helps eliminate cellular waste and maintain mitochondrial function [49,51].

#### 4.2.2. Detrimental effects

Despite its protective aspects, excessive ER stress in nephrotoxic AKI can lead to apoptosis, primarily through CHOP activation. Overexpression of CHOP increases oxidative stress and mitochondrial impairment, promoting cell apoptosis [57,58]. Excessive activation of the IRE1 pathway also interacts with caspase-12, initiating an apoptotic cascade [59]. Additionally, ER stress-induced calcium release activates enzymes like calpain and caspase-12, triggering mitochondrial apoptosis pathways. Prolonged ER stress may also lead to excessive autophagy, which impairs cellular function and contributes to cell death [42,60].

Recognizing the dual roles of ER stress in nephrotoxic AKI highlights the potential for therapeutic strategies that modulate ER stress. Future treatments could focus on balancing ER stress to support adaptive responses while suppressing pro-apoptotic and pro-inflammatory pathways, thus protecting renal function (Table 1) [61–75].

**Table 1.** The protective effects of ER stress on AKI.

AKI	Protective effects	Mechanisms
IRI-AKI	Protein folding assistance [61]	Activating PERK and ATF6 pathways
	Reducing ER burden [62]	Phosphorylation of eIF2α *via* the PERK pathway
	Anti-inflammatory [63]	Inhibition of NF-κB signaling pathway by activating ATF6 pathway
	Anti-oxidative stress [64]	PERK pathway activation induces Nrf2 expression
	Cytoprotection and repair [65]	Promote the expression of cell cycle proteins and growth factors
	Mitigation of apoptosis [66]	Inhibit the Bax/Bak pathway
	Promoting angiogenesis [67]	Increasing the expression of growth factors, such as VEGF
	Autophagy regulation [68]	Induced the expression of autophagy-related genes
Nephrotoxic-AKI	Protein folding assistance [69]	Through the intervention of molecular chaperones such as PDIs and HSPs
	Alleviating ER burden [70]	Activate protein degradation pathways, such as the ERAD pathway
	Anti-inflammatory [71]	Inhibit NF-κB signaling pathway and regulate the expression of anti-inflammatory factors
	Anti-oxidative stress [72]	Nrf2/ARE is activated to induce the expression of antioxidant enzymes
	Cytoprotection and repair [73]	Promote the expression of cell cycle proteins and growth factors
	Benign renal remodeling [74]	Regulate the expression of MMP and TIMP in renal tubular epithelial cells
	Maintenance of cellular homeostasis [75]	Activation of autophagy and mitochondrial quality control pathways

*Abbreviations:* AKI: Acute Kidney Injury; IRI-AKI: Ischemia-Reperfusion Injury Acute Kidney Injury; PERK: Protein Kinase RNA-like Endoplasmic Reticulum Kinase; ATF6: Activating Transcription Factor 6; eIF2α: Eukaryotic Initiation Factor 2 Alpha; NF-κB: Nuclear Factor Kappa B; Nrf2: Nuclear Factor Erythroid 2Related Factor 2; Bax/Bak: Bcl-2 Associated X protein/Bcl-2 Antagonist Killer; VEGF: Vascular Endothelial Growth Factor; PDIs: Protein Disulfide Isomerases; HSPs: Heat Shock Proteins; ERAD: Endoplasmic Reticulum-Associated Degradation; ARE: Antioxidant Response Element; MMP:Matrix Metalloproteinases; TIMP: Tissue Inhibitors of Metalloproteinases.

## 5. Targeting ER homeostasis: a novel therapeutic strategy for AKI

Given the complex role of ER stress in both promoting cell survival and exacerbating cell death in AKI, targeting ER homeostasis presents a dual approach to mitigate damage while enhancing cellular repair mechanisms.

Targeting ER homeostasis for drug intervention offers a promising therapeutic avenue for AKI. In the context of AKI, ER stress is typically exacerbated due to disturbances in protein folding and accumulation of unfolded proteins, potentially leading to cellular dysfunction and death. Modulating the ER stress response can restore ER function, protect renal cells from damage, and thereby mitigate the severity of AKI.Some studies have focused on exploring the role of chemical chaperones like 4-phenylbutyric acid (4-PBA) and tauroursodeoxycholic acid (TUDCA) [76,77]. These chaperones assist in the proper folding of proteins, reducing the accumulation of unfolded proteins in the ER and alleviating ER stress. In AKI models, these compounds have shown the potential to protect renal cells from injury, suggesting their viability as effective drugs for AKI treatment. On the other hand, small molecule inhibitors targeting ER stress pathways have also been investigated as therapeutic strategies for AKI. For instance, inhibitors of IRE1α [78] can prevent the overactivation of ER stress signaling, thus protecting cells from death. Similarly, inhibition of the PERK pathway has shown potential in preventing AKI by avoiding excessive translational inhibition and cellular stress responses [58,79]. Moreover, drugs that promote autophagy have also demonstrated potential in the treatment of AKI. Autophagy is an intracellular degradation pathway that helps to remove damaged ER and other organelles [80], thereby reducing ER stress. By activating autophagy, cellular tolerance to ER stress can be enhanced, lessening the impact of AKI. However, although these strategies show promise in preclinical models, their safety and efficacy need to be further validated in clinical trials. Additionally, drug interventions targeting ER homeostasis may require customization based on the specific causes and stages of AKI to maximize therapeutic effects(Table 2).

**Table 2.** Potential pharmacological interventions targeting ER homeostasis in AKI.

Type of compound/drug	Mechanism of action	Research phase	Potential effects
Chemical Chaperones (4-PBA, TUDCA)	Assist in proper protein folding, reduce accumulation of unfolded proteins in the ER	Preclinical	Alleviate ER stress, protect renal cells
IRE1α Inhibitors	Prevent overactivation of ER stress signaling	Preclinical	Reduce programmed cell death
PERK Pathway Inhibitors	Avoid excessive translational inhibition and cellular stress responses	Preclinical	Prevent AKI
Autophagy-Promoting Drugs	Enhance cellular tolerance to ER stress, clear damaged ER	Preclinical/Early Clinical	Mitigate the impact of AKI

*Abbreviations:* AKI: Acute Kidney Injury; ER: endoplasmic reticulum; 4-PBA: 4-phenylbutyric acid; TUDCA: tauroursodeoxycholic acid; IRE1α: Inositol-Requiring Enzyme 1α; PERK: Protein Kinase RNA-like Endoplasmic Reticulum Kinase.

In summary, drug interventions targeting ER homeostasis present a new therapeutic strategy with the potential to improve outcomes for patients with AKI. Future research will need to delve deeper into the specific mechanisms of these interventions and how these findings can be translated into effective treatments in clinical practice.

## 6. Interactions between ER stress and other cellular processes in AKI

ER stress interacts closely with various cellular processes, including inflammation, apoptosis, and autophagy, and these interactions play a pivotal role in the progression of AKI. Recent studies have illuminated how ER stress contributes to AKI pathogenesis by modulating these processes and impacting cellular homeostasis. Understanding these interactions is essential for developing targeted therapies with improved efficacy and minimized adverse effects.

### 6.1. ER stress and inflammation

ER stress is known to be both a contributor to and a consequence of inflammation in AKI. The IRE1 and PERK pathways play dual roles in modulating inflammatory responses. For example, IRE1 activation leads to NF-κB pathway stimulation, which increases the production of pro-inflammatory cytokines such as TNF-α, IL-1β, and IL-6. While this response can help recruit immune cells to injury sites, chronic activation may exacerbate renal injury through sustained inflammation [81]. Conversely, the ATF6 pathway has been shown to inhibit the NF-κB pathway under moderate ER stress, reducing cytokine release and inflammation. This anti-inflammatory function highlights the potential of selective pathway modulation to reduce inflammation in AKI without impairing necessary immune responses [82].

### 6.2. ER stress and apoptosis

ER stress-induced apoptosis is primarily mediated through CHOP activation in the PERK pathway. CHOP promotes apoptosis by upregulating pro-apoptotic proteins (e.g., Bax and Bak) and inhibiting anti-apoptotic proteins like Bcl-2, which leads to mitochondrial dysfunction and cell death [83]. IRE1 can also contribute to apoptosis through the activation of JNK, which further promotes pro-apoptotic signaling cascades [84]. In the context of AKI, apoptosis serves as a mechanism to eliminate irreversibly damaged renal cells. However, excessive apoptosis can impair kidney function and hinder recovery, making it essential to balance ER stress-mediated apoptosis to avoid exacerbating injury.

### 6.3. ER stress and autophagy

Autophagy is a protective mechanism that clears damaged organelles and misfolded proteins, thus reducing ER stress and promoting cellular recovery. Under moderate stress, ER stress induces autophagy through pathways like IRE1 and PERK, which help cells adapt to adverse conditions [85]. However, prolonged ER stress may lead to autophagic dysregulation, resulting in excessive autophagy that disrupts cellular homeostasis and promotes cell death [86]. This dual role of autophagy suggests that therapies enhancing adaptive autophagy, while preventing excessive autophagy, may be beneficial for treating AKI.

Understanding the crosstalk between ER stress, inflammation, apoptosis, and autophagy emphasizes the need for therapies that precisely target these interactions to mitigate renal injury without compromising cellular recovery mechanisms.

## 7. Limitations of current therapies and preclinical models for ER stress modulation in AKI

Although therapies targeting ER stress pathways hold promise for managing AKI, current approaches face significant limitations. The following critical challenges need to be addressed to improve therapeutic efficacy and safety:

### 7.1. Off-target effects and specificity issues

ER stress modulators often lack specificity, leading to unintended effects on other cellular processes. For instance, while some modulators may reduce ER stress, they might inadvertently suppress essential UPR pathways required for cell survival. Furthermore, therapies targeting specific pathways like PERK or IRE1 may have limited efficacy across different AKI types due to variability in ER stress responses. Therefore, developing selective modulators that target specific ER stress pathways involved in renal protection, while sparing those pathways needed for cellular adaptation, is essential [87].

### 7.2. Balancing protective and harmful ER stress responses

ER stress plays both protective and harmful roles, making it challenging to achieve a balance that maximizes therapeutic benefits. Over-suppressing ER stress may hinder necessary adaptive responses, while excessive activation could lead to apoptosis and inflammation. Thus, the therapeutic approach must carefully modulate ER stress to maintain a balance between adaptation and cell death. Current research suggests that dynamic modulation—adjusting therapy based on the stage and severity of AKI—could help optimize this balance, yet it remains a challenging goal to achieve in clinical settings [88].

### 7.3. Limitations in preclinical models

Preclinical models, while invaluable for understanding ER stress in AKI, have limitations that may restrict the translation of findings to human applications. Most models use acute injury conditions that do not fully represent the complexity of human AKI, particularly in cases of chronic kidney injury. Additionally, animal models may exhibit differences in ER stress responses compared to human tissues, complicating the prediction of clinical outcomes. Refining preclinical models to better simulate human AKI conditions and incorporating models that mimic chronic injury are crucial for the development of effective ER stress-targeted therapies [89].

In conclusion, while ER stress-targeting therapies hold significant potential, further advancements are needed to overcome these limitations. Future research should focus on improving pathway specificity, achieving optimal stress balance, and refining preclinical models to ensure that findings translate effectively into clinical practice.

## 8. Conclusions

ER stress plays a significant role in AKI. It can both exacerbate kidney injury through the induction of apoptosis, inflammation, and oxidative stress, and attenuate AKI through cytoprotective and repair mechanisms, such as autophagy, antioxidative stress, and inflammatory modulation. Researchers have identified several ER stress-related targets, like PERK, IRE1, and ATF6, which can influence AKI by regulating the UPR signaling pathway. Studying these targets may lead to new therapeutic strategies. Progress has been made in developing drugs targeting ER stress, such as chemical chaperones like TUDCA and 4-PBA, which have been shown to reduce ER stress and protect kidney function. Additionally, inhibitors or activators targeting specific ER stress signaling pathways are under investigation for their potential application in AKI treatment. However, numerous challenges and unresolved issues remain. The interactions and regulatory mechanisms between ER stress and other signaling pathways (e.g., NF-κB, MAPK, Nrf2) are not yet fully understood, and a deeper comprehension of these interactions could help in discovering new therapeutic strategies. Developing drugs that target ER stress effectively, while exhibiting better targeting and selectivity to avoid adverse effects on normal cells and tissues, is crucial. Individual differences among AKI patients may necessitate precision treatment and careful consideration of safety issues. With a more comprehensive understanding of ER stress mechanisms and advancements in research techniques, we anticipate more effective treatment options for AKI in the future.
